# LAUNCHING A NEW UNIVERSITY-BASED OPENING MINDS THROUGH ARTS PROGRAM

**DOI:** 10.1093/geroni/igae098.1506

**Published:** 2024-12-31

**Authors:** Dawn Carr, Suzanne Smith Monroe

**Affiliations:** Florida State University, Tallahassee, Florida, United States; Claude Pepper Center, Tallahassee, Florida, United States

## Abstract

Florida State University launched the Opening Minds through Arts program in Fall of 2023 within the college of Social Sciences and Public Policy. The current model is based in a student internship program, in which students are able to earn up to three credit hours. The internship course includes one face-to-face, hour-long class per week with an instructor in which they complete course readings, watch videos, and listen to podcasts to learn about Alzheimer’s and Dementia related subjects, as well as research associated with art-based therapies and person-centered care models. Students are paired with one older adult for the semester at one of our two partnership residential care homes, in which students facilitate art making and, in the process, get to know their older adult artist. Since the program’s launch, FSU has trained 18 student interns and 13 volunteers from varying disciplines, such as the FSU College of Law. The program has also served 19 older adults in Tallahassee and continues its plan to expand the number of OMA sessions, with the Spring semester currently holding three OMA sessions per week (5 older adults per session). Although FSU’s program is in early stages of development, we will provide internal program evaluation results which includes institutional partnership characteristics that promote long-term success, student training and preparation, and qualitative feedback from student interns. We will also provide information about expansion of the program for clinical settings at FSU to expand geriatric training and person-centered care.

